# Carotid endarterectomy for treatment of tandem carotid stenosis in the presence of the anomalous origin of the occipital artery arising from the cervical internal carotid artery: a case report

**DOI:** 10.1186/1752-1947-7-254

**Published:** 2013-11-07

**Authors:** Gakushi Yoshikawa, Mariko Kawashima, Kazuo Tsutsumi

**Affiliations:** 1 Department of Neurosurgery, Showa General Hospital, 8-1-1 Hanakoganei, Kodaira City, Tokyo 187-8510, Japan

**Keywords:** Anomalous branch, Carotid endarterectomy, Tandem stenosis

## Abstract

**Introduction:**

Branches from the cervical portion of the internal carotid artery are rare. In most cases, atherosclerotic stenosis is found at the bifurcation of the internal and external carotid arteries. However, when associated with atherosclerotic carotid artery disease, the origin of the rare branches arising from the internal carotid artery can be another site of stenosis. This report describes a rare case of such tandem carotid stenosis treated by carotid endarterectomy and the importance of the possibility of stenosis at the origin of the anomalous branch from the internal carotid artery.

**Case presentation:**

A 73-year-old Japanese woman presented with transient left hemiparesis and vertigo. Magnetic resonance angiography seemed to indicate two stenotic lesions distal to the right internal carotid artery in addition to the origin of the right internal carotid artery, and angiography indicated tandem stenotic lesions of the internal carotid artery. The patient was successfully treated with right carotid endarterectomy, including the distal stenotic lesion of internal carotid artery, and postoperative angiography indicated that the occipital artery arose from the internal carotid artery.

**Conclusion:**

It is important to recognize rare cases of the anomalous origin of the occipital artery from the internal carotid artery and the possibility that the origin of such an anomalous occipital artery may be the cause of stenosis.

## Introduction

Branches of the extracranial portion of the internal carotid artery (ICA) are rare. Although atherosclerotic stenosis is commonly found at the origin of the ICA from the common carotid artery, when associated with atherosclerotic carotid disease, the origin of the rare branch from the ICA can be another site of atherosclerotic stenosis. In this report, we describe the case of a patient with symptomatic tandem atheromatous plaques of the ICA located not only at the common stenotic site of the origin of the ICA but also at the anomalous origin of the occipital artery from the ICA.

## Case presentation

**Figure 1:**
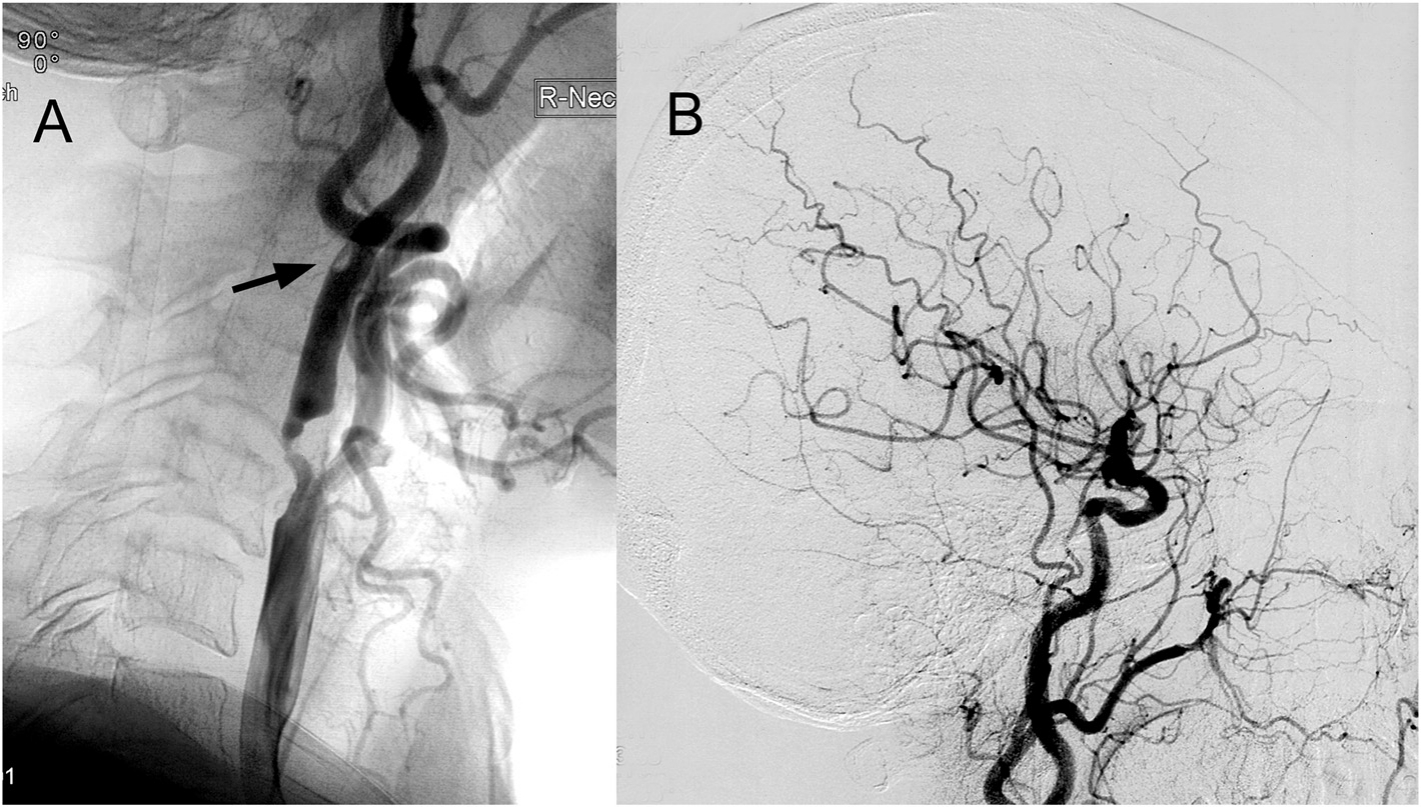
**Lateral projection of preoperative right carotid angiogram.** These images show atherosclerotic stenosis not only at the origin of the internal carotid artery but also distal to the internal carotid artery **(A)** (arrow) and poor filling of the occipital artery because of stenosis at the origin from the internal carotid artery **(B)**.

A 73-year-old Japanese woman presented with a recent episode of vertigo and transient left upper-extremity weakness and numbness. The patient had only hypertension and no history of coronary artery disease, diabetes or smoking. Her neurological examination revealed no remarkable findings. Magnetic resonance imaging demonstrated two stenotic lesions at the distal right ICA in addition to the origin of the ICA but no evidence of cerebral infarction. Carotid angiography revealed tandem stenotic lesions not only at the origin of the ICA but also distal to the ICA (Figures 1A and 1B).

**Figure 2:**
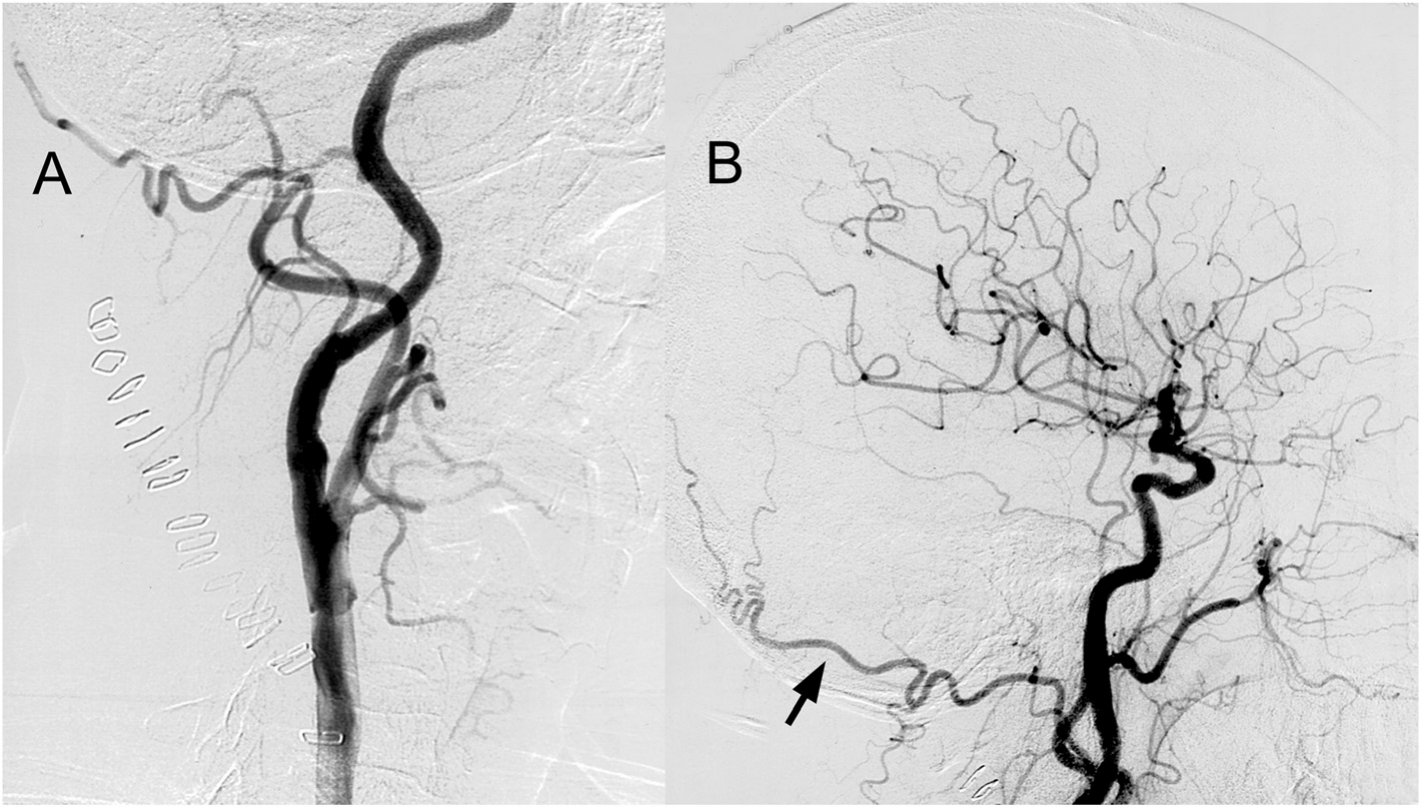
**Lateral projection of postoperative right carotid angiogram.** These images show complete revascularization both of the stenotic lesions of the internal carotid artery **(A)** and demonstrate good filling of the occipital artery **(B)** (arrow).

Right carotid endarterectomy was performed with carotid exposure to the end of the distal plaque, and an anomalous branch from the ICA was found. During the procedure, the shunt tube was inserted to provide bypass blood flow to the right ICA distally. Post-operatively, the patient remained neurologically intact, and angiography revealed that the anomalous branch from the ICA at the distal stenotic site was the occipital artery (Figures 2A and 2B). Pathological examination of the plaque revealed marked atherosclerosis with calcification.

## Discussion

Anomalous branches of the cervical portion of the ICA are rare. In 1968, Newton and Young reported the cases of three patients, all of whom had an occipital artery arising from the ICA distal to the bifurcation [1]. In the 1970s, there were a few other reports of anomalous origin of the occipital artery from the ICA [2,3]. Benton *et al.* reported a case in which carotid endarterectomy was performed to remove the atheromatous plaque at the origin of the ICA, and, during the procedure, the anomalous origin of the occipital artery was found to bifurcate 2cm distal to the origin of the ICA [4]. The other anomalous vessels arising from the ICA, which are present during fetal development, known as *persistent carotid-basilar anastomoses*, have been described in relation to atherosclerotic cerebrovascular disease as the cause of the ICA stenosis [5-13]. There have been no reports that the origin of anomalous branches from the ICA are involved in atherosclerotic stenosis.

Atherosclerosis is strongly associated with carotid intimal thickness, which is in fact a complex process dependent on a variety of factors. Among those factors, local hemodynamics, such as high blood pressure and sheer stress, turbulent flow and subsequent intimal injury, play an important role. Mechanisms such as those described above may explain at least in part why atherosclerosis commonly develops at the vascular branching points, especially why the origin of the ICA from the common carotid artery is the most common site for atherosclerotic stenosis.

In this case, we supposed that atherosclerotic change might develop at the common site of the carotid artery, and the plaque extended to the distal and the unusual location branching anomalous occipital artery was involved to form the atherosclerotic plaque. As branches of the extracranial portion of the ICA are rare and most of the unusual branches have been demonstrated by incidental angiography during the investigation of cerebral aneurysms or other vascular malformations, when the apparent atherosclerotic plaque is found at the origin of the ICA, the distal plaque of the ICA may be missed. Considering the close origin of the ICA, however, it is likely that the anomalous origin of the branch from the ICA can become involved with atherosclerotic plaque.

## Conclusions

Recognition of the possibility of tandem atherosclerotic lesions, not only at the origin of the ICA of the common stenotic site but also at the anomalous origin of the branch from the ICA, would be helpful for proper management during carotid endarterectomy. Moreover, a careful review of preoperative angiography is important.

## Consent

Written informed consent was obtained from the patient for publication of this case report and any accompanying images. A copy of the written consent is available for review by the Editor-in-Chief of this journal.

## Competing interests

The authors declared that they have no competing interests.

## Authors’ contributions

GY and MK performed the surgery and clinical evaluation of the patient. GY, MK and KT analyzed and reviewed all examinations and the medical history of the patient regarding pathology. All authors read and approved the final manuscript.
